# Exploring Pathways for Pain Relief in Treatment and Management of Lumbar Foraminal Stenosis: A Review of the Literature

**DOI:** 10.3390/brainsci14080740

**Published:** 2024-07-24

**Authors:** Renat Nurmukhametov, Manuel De Jesus Encarnacion Ramirez, Medet Dosanov, Abakirov Medetbek, Stepan Kudryakov, Gervith Reyes Soto, Claudia B. Ponce Espinoza, Jeff Natalaja Mukengeshay, Tshiunza Mpoyi Cherubin, Vladimir Nikolenko, Artem Gushcha, Salman Sharif, Nicola Montemurro

**Affiliations:** 1 2nd National Clinical Centre, Federal State Budgetary Research Institution, Russian Research Center of Surgery Named after Academician B.V. Petrovsky, 103274 Moscow, Russia; 2 Department of Neurosurgery, Russian People’s Friendship University, 121359 Moscow, Russia; 3 Department of Neurosurgery, Sechenov First Moscow State Medical University (Sechenov University), Ministry of Health of the Russian Federation, 103220 Moscow, Russia; 4 Department of Head and Neck, Unidad de Neurociencias, Instituto Nacional de Cancerología, Mexico City 14080, Mexico; 5 Clinique Ngaliema, Kinshasa 7948, Democratic Republic of the Congo; 6 Department of Neurosurgery, Research Center of Neurology, 103220 Moscow, Russia; 7 Department of Neurosurgery, Liaquat National Hospital and Medical College, Karachi 16250, Pakistan; 8 Department of Neurosurgery, Azienda Ospedaliero Universitaria Pisana (AOUP), 56100 Pisa, Italy

**Keywords:** lumbar foraminal stenosis, neurosurgery, microsurgical skills, surgical treatment, surgical outcome

## Abstract

Background: Lumbar foraminal stenosis (LFS) involves the narrowing of neural foramina, leading to nerve compression, significant lower back pain and radiculopathy, particularly in the aging population. Management includes physical therapy, medications and potentially invasive surgeries such as foraminotomy. Advances in diagnostic and treatment strategies are essential due to LFS’s complexity and prevalence, which underscores the importance of a multidisciplinary approach in optimizing patient outcomes. Method: This literature review on LFS employed a systematic methodology to gather and synthesize recent scientific data. A comprehensive search was conducted across PubMed, Scopus and Cochrane Library databases using specific keywords related to LFS. The search, restricted to English language articles from 1 January 2000 to 31 December 2023, focused on peer-reviewed articles, clinical trials and reviews. Due to the heterogeneity among the studies, data were qualitatively synthesized into themes related to diagnosis, treatment and pathophysiology. Results: This literature review on LFS analyzed 972 articles initially identified, from which 540 remained after removing duplicates. Following a rigorous screening process, 20 peer-reviewed articles met the inclusion criteria and were reviewed. These studies primarily focused on evaluating the diagnostic accuracy, treatment efficacy and pathophysiological insights into LFS. Conclusion: The comprehensive review underscores the necessity for precise diagnostic and management strategies for LFS, highlighting the role of a multidisciplinary approach and the utility of a unified classification system in enhancing patient outcomes in the face of this condition’s increasing prevalence.

## 1. Introduction

Lumbar foraminal stenosis (LFS) is a critical pathological condition characterized by the narrowing of the neural foramina, resulting in potential nerve root compression that often leads to significant lower back pain and radiculopathy [1]. This condition poses a significant burden on healthcare systems worldwide, with a prevalence that increases with the aging of the population [1]. As the spinal structures undergo degenerative changes, the risk of LFS escalates, necessitating advancements in both diagnostic and therapeutic strategies [1,2]. The complexity of LFS, influenced by both biomechanical factors and individual patient characteristics, requires a nuanced understanding of its pathophysiology, diagnostic criteria and management options to optimize patient outcomes [3]. The etiology of LFS is multifactorial, involving both acquired and congenital factors [3]. Degenerative changes such as disc herniation, spondylolisthesis and osteophyte formation are among the predominant causes [3]. These pathological changes contribute to a reduction in foraminal space, impinging on nerve roots and leading to clinical symptoms that significantly impair patients’ quality of life [4,5]. The symptoms associated with LFS, including radicular leg pain, numbness at related dermatome levels and muscle weakness, often mimic those of other neuromusculoskeletal disorders, which can complicate diagnosis and delay appropriate treatment [6].

Current diagnostic modalities for LFS incorporate a combination of clinical evaluation, imaging techniques such as MRI and CT scans and sometimes diagnostic nerve blocks. Both MRI and CT scans provide unique insights into the anatomical aspects of the spine, but also present limitations in terms of sensitivity, specificity and accessibility [7,8]. This highlights the need for the continuous refinement of diagnostic criteria and the development of new technologies that can provide more accurate and timely diagnoses [9]. The grading of foraminal stenosis based on imaging findings, particularly MRI, plays a crucial role in the diagnostic process, helping to quantify the extent of pathological changes and guide treatment decisions. However, the variability in individual anatomy and the potential for radiographic findings to overestimate or underestimate the degree of nerve compression necessitate a judicious interpretation of these results [8]. To accurately diagnose and manage leg pain resembling symptoms of foraminal neuropathy, distinguishing between various conditions is essential. Conditions such as radiculopathy, resulting from nerve root compression due to spinal stenosis or disc herniation, present sharp, radiating pain along the nerve’s course. Extraforaminal disorders, affecting nerves exiting the spine, mimic foraminal neuropathy but originate outside the spinal canal. 

The management of LFS typically involves a multidisciplinary approach, ranging from conservative treatments like physical therapy and pharmacotherapy to more invasive options such as epidural steroid injections, pulsed radiofrequency and surgical interventions [10,11]. Conservative management often serves as the first line of treatment, aiming to alleviate pain and improve functional status without the risks associated with surgery. However, for patients with severe or persistent symptoms, surgical options such as foraminotomy or laminectomy may be considered [12,13].

While imaging techniques such as MRI and CT myelography offer detailed insights into the pathological changes underlying foraminal stenosis, their findings must be carefully correlated with the patient’s clinical presentation. Radiographic evidence of spinal degeneration, including disc space narrowing and facet joint hypertrophy, is common even in asymptomatic individuals, underscoring the importance of a comprehensive diagnostic approach that integrates patient history, physical examination and the selective use of imaging and electrodiagnostic tests [9,11].

This literature review seeks to encapsulate the current knowledge and recent advancements in the diagnosis and management of LFS. By examining a wide array of peer-reviewed studies, clinical trials and meta-analyses, this review aims to synthesize the findings into a coherent framework that can guide future research and clinical practice. In doing so, it will address the critical gaps in the existing literature and suggest areas where further investigation is needed. 

The aim of this review is to contribute to improving the knowledge of all possible pathways to relieve pain and improve clinical outcomes for patients with LFS. As the prevalence of LFS continues to rise in line with an aging global population, the importance of such a comprehensive review becomes increasingly important, serving not only as a resource for healthcare professionals but also as a roadmap for future research endeavors.

## 2. Materials and Methods

This literature review was conducted following a methodology designed to gather and analyze the most relevant and recent scientific data on LFS. The purpose of this approach is to ensure a comprehensive synthesis of the information, which allows for an in-depth understanding of the current diagnostic and therapeutic advancements in the management of LFS.

### 2.1. Literature Search Strategy

To capture a broad spectrum of relevant literature, a search was conducted across PubMed, Scopus and Cochrane Library databases. The search was performed using a combination of keywords and MeSH terms to ensure completeness. The terms included “lumbar foraminal stenosis”, “spinal stenosis”, “foraminal narrowing”, “nerve root compression”, and related procedural keywords like “foraminotomy”, “laminectomy”, and “epidural steroid injection”. The search was limited to articles published in the English language between 1 January 2000 and 31 December 2023. This time frame was chosen to ensure that the review included both foundational studies and the most current research developments. Additional filters were applied to include only peer-reviewed articles, clinical trials, meta-analyses and systematic reviews to ensure the quality and reliability of the included studies. As this is a literature review, we did not register this paper in PROSPERO.

### 2.2. Study Selection

The initial search results were independently screened by two reviewers (R.N. and M.E) based on title and abstract relevance. Articles that met the preliminary inclusion criteria were retrieved in full text for a detailed evaluation. The inclusion criteria were (1) studies that focused specifically on LFS, (2) studies that provided original data or analyses on diagnostic methods, treatment outcomes or pathophysiological insights into LFS, and (3) studies that evaluated both conservative and surgical management approaches. The exclusion criteria included (1) studies focusing on non-lumbar spinal stenosis, commentaries, editorials and opinion pieces without original data, and (2) studies with incomplete data or lacking peer review. Any discrepancies between reviewers during the selection process were resolved through discussion or consultation with a third reviewer (N.M.).

### 2.3. Data Extraction

Data from the selected studies were extracted using a standardized form, which included the following information: study design and methodology, demographics and baseline characteristics, details of diagnostic procedures used, treatments evaluated and their outcomes, key findings related to the pathophysiology of LFS and type of foraminal stenosis.

### 2.4. Quality Assessment, Data Synthesis and Analysis

The quality of the included studies was assessed using established checklists adapted from the PRISMA guidelines [12]. Assessment criteria included the clarity of data presentation, the appropriateness of the study design for the research question, the risk of bias and the impact of the study findings on the field. The data were synthesized qualitatively due to the heterogeneity in the study designs, populations, interventions and outcomes. The findings were organized into thematic categories corresponding to the diagnosis, treatment and pathophysiological mechanisms of LFS. A narrative synthesis approach was used to integrate findings across different studies, highlighting both consistencies and discrepancies. This methodological framework provided a comprehensive review of the literature on LFS, aiming to offer a clear and scientific synthesis of the available data to guide future research and clinical practice. The results are expected to inform both clinical decision making and policy development in the management of LFS.

## 3. Results

**Figure 1:**
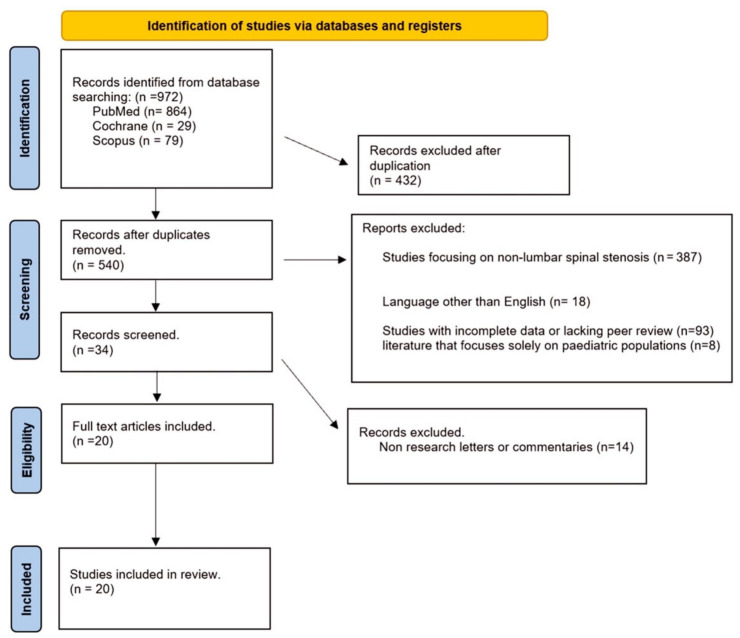
PRISMA flow diagram.

A total of 972 articles were identified and all abstracts were reviewed, according to PRISMA guidelines (Figure 1). Following the removal of duplicates, 540 articles remained. After screening titles and abstracts, articles were excluded as they did not meet the inclusion criteria and 34 full-text articles were assessed for eligibility. Subsequently, a full-text review of 20 articles was performed and finally 20 articles were included [13,14,15,16,17,18,19,20,21,22,23,24,25,26,27,28,29,30,31,32]. Table 1, Table 2 and Table 3 show all the details.

## 4. Discussion

**Figure 2:**
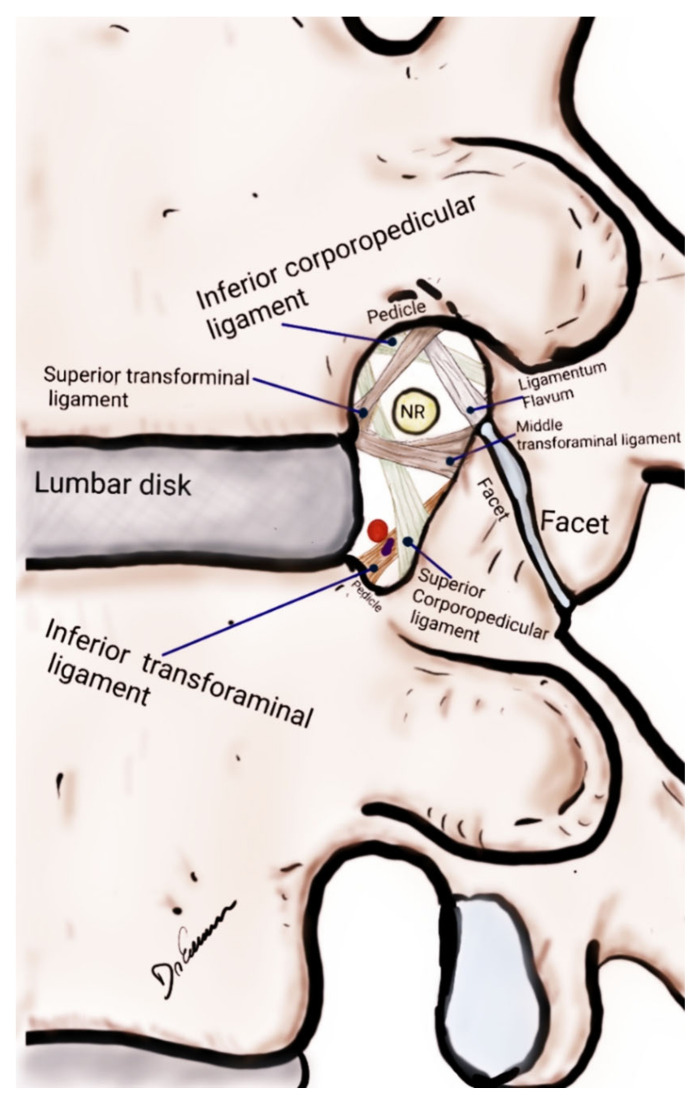
Drawing of a lateral view of the lumbar spine focused on the anatomical structures of the lumbar foraminal space, lumbar disc and facet joint. NR, nerve root.

A comprehensive understanding of LFS pathophysiology is critical not only for accurate diagnosis but also for the effective management of the condition (Figure 2). The etiology of LFS is multifactorial, encompassing both congenital and acquired factors. Disc herniation and degenerative changes such as spondylolisthesis and the formation of osteophytes are the most common causes [35,36].

### 4.1. Diagnostic Challenges and Considerations in LFS

**Figure 3:**
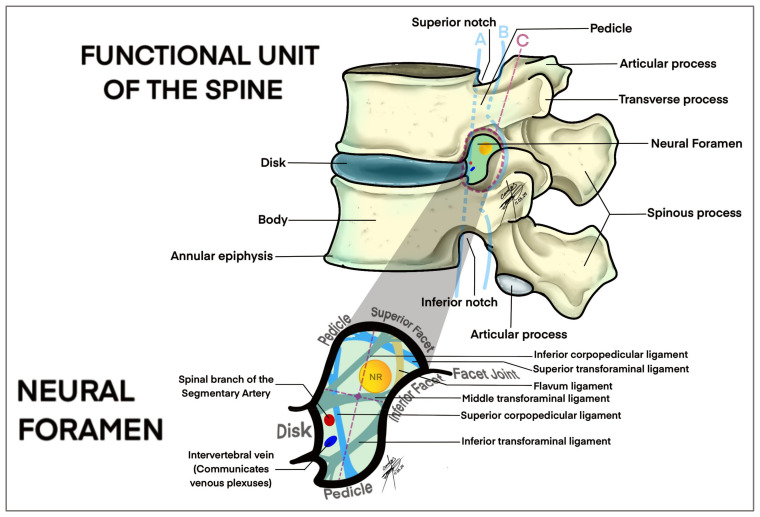
Drawing of a lateral view of the lumbar spine focused on the anatomical structures of the lumbar disc, ligaments, facet joints and vascular structures related with lumbar foraminal space (intraforaminal, foraminal, extraforaminal). A, intraforaminal; B, foraminal; C, extraforaminal.

The diagnostic process for LFS usually begins with a thorough clinical evaluation, including a detailed history and physical examination. Symptoms such as radicular pain, numbness and muscle weakness are typical indicators of nerve root compression due to foraminal narrowing. However, these symptoms can overlap with other spinal pathologies, making clinical evaluation alone insufficient for a definitive diagnosis [7,8]. Kaneko et al. [20] and Lorenc et al. [32] highlighted the significance of MRI as the most sensitive imaging modality for diagnosing LFS. It provides detailed images of both bone and soft tissues, including nerves, muscles, ligaments and intervertebral discs. The role of disc, neuronal foramen, facet joints and ligaments and their relationship with the nerve root can be better understood by looking at Figure 3.

MRI is particularly useful for visualizing the extent of nerve root compression and the condition of soft tissues, which can indicate inflammation or other pathological changes associated with stenosis [14,16]. Its non-invasive nature and absence of ionizing radiation make it suitable for repeated follow-up assessments. However, MRI’s sensitivity means it may also reveal abnormalities that are asymptomatic or clinically insignificant, which can sometimes lead to overdiagnosis or overtreatment [36]. On the other hand, artifacts generated by screws after surgery remain a significant problem in the MRI of the lumbar spine [36]. CT scans are less sensitive than MRI ones for soft tissue visualization but are more specific for detecting bony changes such as osteophytes and facet joint hypertrophy, which are common causes of foraminal narrowing [37]. When combined with myelography (CT myelography), where a contrast dye is injected into the spinal canal, CT can provide a clear picture of how the spinal cord and nerve roots are affected by the bony structures. This combination enhances both the sensitivity and specificity of the CT scan, making it particularly useful in patients who cannot undergo MRI, such as those with pacemakers or other metallic implants [23,24,30].

While not a primary diagnostic tool for LFS, electrodiagnostic tests such as electromyography (EMG) and nerve conduction studies can be used to confirm nerve impairment and assess its severity [37]. These tests are specific but less sensitive; they are typically employed to rule out other causes of neuropathy or to confirm a diagnosis in complex cases where imaging results are inconclusive [38]. Classifying LFS is essential for guiding treatment strategies and ensuring consistent communication among clinicians, encompassing etiology, anatomical location and severity [14,15,16].

The etiological classification of LFS includes degenerative, the most common type, resulting from age-related changes like facet joint osteoarthritis, disc degeneration and ligament thickening [25]. Congenital variations may predispose some individuals to narrower foramina, while iatrogenic stenosis can occur following surgical interventions that alter spinal anatomy or cause scarring [39,40,41]. Traumatic stenosis results from injuries that disrupt spinal alignment, such as fractures or dislocations [25,28].

**Figure 4:**
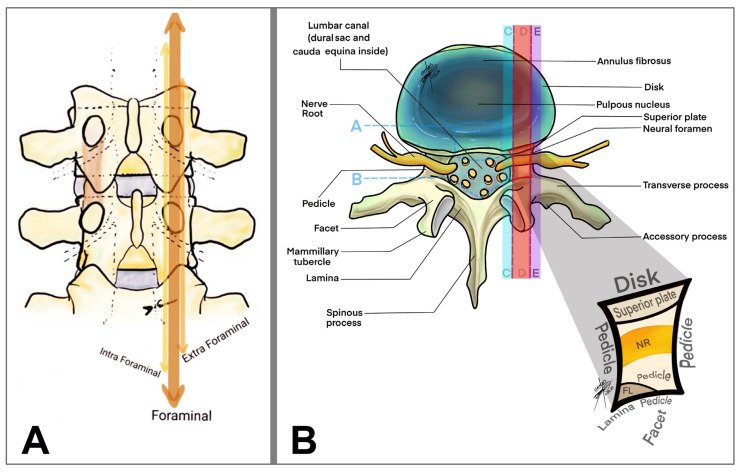
Drawing of a coronal (**A**) and axial (**B**) view of the lumbar spine focused on the lumbar foraminal space and its anatomical division (arrows in (**A**) and colorful stripes in (**B**)): A, anulus; B, dural sac; C, intraforaminal; D, foraminal; E, extraforaminal; NR, nerve root.

Anatomically, LFS can be classified as (1) lateral (intraforaminal), where the stenosis occurs within the boundaries of the foramen; (2) far lateral (extraforaminal), affecting the area outside the spinal canal but impacting exiting nerve roots and (3) central, involving primarily the central spinal canal but contributing indirectly to foraminal narrowing through mechanisms like the hypertrophy of the ligamentum flavum or disc protrusions [15,31,32] (Figure 4).

Severity-based classification uses imaging-based grading systems, crucial for diagnosis and evaluating treatment efficacy. Mild LFS shows foraminal narrowing without evident nerve root impingement and may be asymptomatic or cause mild symptoms [42]. Moderate LFS, with evident changes in the foramen and nerve roots entrapment, often correlates with symptoms such as radiculopathy. Severe LFS involves significant narrowing with clear compression of the nerve roots and substantial loss of perineural fat, typically associated with severe pain and neurological deficits [43]. These classifications guide clinicians in tailoring treatment strategies (from conservative management for mild cases to potential surgical interventions for severe instances) and in predicting prognosis. They also enhance communication among healthcare providers by providing a clear, standardized description based on clinical and radiographic criteria. Furthermore, these systems facilitate clinical research by enabling comparisons across studies and aiding in the development of management guidelines [44,45,46].

### 4.2. Evolution of Treatment Paradigms

The management of LFS has undergone significant transformation over recent years, transitioning from conventional conservative methods to more sophisticated, personalized surgical interventions that are tailored to the specific anatomical and pathological characteristics of individual patients. Minimally Invasive Surgery (MIS) techniques, such as minimally invasive transforaminal lumbar interbody fusion (MIS-TLIF), have emerged as pivotal advancements in the treatment of this condition. Liu et al. [15] demonstrated the efficacy of MIS-TLIF for effectively managing foraminal stenosis with minimal invasiveness, particularly highlighting its suitability for unilateral symptoms that do not necessitate extensive bilateral decompression. This approach not only alleviates symptoms but also promotes a quicker recovery by reducing surgical morbidity compared to more traditional open surgeries [19,21].

However, the adoption of unilateral procedures like those studied by Chen et al. [18] brings to light potential complications such as contralateral radiculopathy, underscoring the need for meticulous surgical planning and technique refinement. This includes the importance of preoperative planning and intraoperative imaging to precisely place implants and avoid harming contralateral nerve roots, which are essential to minimize postoperative complications and ensure favorable outcomes [47]. Some authors have suggested the use of interspinous spacers or a CT-guided injection focused on nerve roots for the treatment of LFS [48]. Further advancements in surgical strategies have been made through the work of researchers like Özer et al. and Zhu et al., who have focused on optimizing surgical outcomes through precise classifications of stenosis and strategic choices in cage orientation during TLIF procedures [10,30,35]. These studies emphasize a methodical surgical approach that incorporates individual variations in spinal anatomy to enhance the specificity and efficacy of treatments [48]. For example, Özer et al.’s classification system aids surgeons in choosing the most appropriate surgical intervention tailored to the type and severity of stenosis, enhancing predictability in outcomes [28]. Similarly, Zhu et al.’s research on the impact of cage orientation provides crucial insights into how surgical adjustments can significantly influence spinal alignment and foraminal dimensions, which are critical for achieving symptomatic relief and biomechanical stability [15].

The integration of advanced imaging technologies and surgical navigation systems has been pivotal in increasing the precision of these interventions. Real-time imaging facilitates the accurate placement of instruments and implants during procedures, enhancing the safety and effectiveness of the surgery. This dynamic adjustment to intraoperative findings, based on both preoperative imaging and real-time feedback, leads to better alignment with the patient’s specific condition, thereby improving clinical outcomes [49,50]. Looking forward, the evolution of treatment paradigms for LFS is likely to witness further enhancements with the development of advanced biomaterials for spinal implants and the application of artificial intelligence in surgical planning [51,52,53,54]. These innovations promise to refine the personalization of treatment strategies further, potentially leading to the more predictable and effective management of LFS. As such, the field is moving towards a new standard in spinal disorder care, where treatments are not only effective but also minimally disruptive, tailored to individual needs and aligned with the goal of improving patients’ quality of life [55,56,57].

### 4.3. Future Research Directions

Despite significant advancements in both diagnostic and therapeutic fronts, there remains a substantial gap in linking radiological findings with clinical outcomes. Future research should focus on longitudinal studies that track patient outcomes over time to better correlate preoperative imaging findings with postoperative results. Additionally, as highlighted by Haimoto et al., exploring the risk factors for restenosis after surgical interventions could provide insights into long-term management strategies for LFS [23].

### 4.4. Limitation of the Study

Specificity of surgical outcomes: While the article discussed the benefits of new surgical techniques and approaches, there was limited discussion on the long-term outcomes of these surgeries. The impact of these techniques on patient quality of life over time and the durability of symptom relief were not extensively covered.

Generalizability of findings: The findings and recommendations were based on studies with specific patient demographics and clinical settings, which may not have been universally applicable. The variability in surgical success rates, patient recovery and complication rates across different populations and surgical environments was not thoroughly examined.

Economic and accessibility considerations: The article did not address the economic implications of advanced surgical techniques and imaging technologies. The availability and affordability of these innovations in different healthcare systems, particularly in lower-resource settings, are important aspects that were not discussed.

Comparative analysis: There was a lack of detailed comparative analysis between different surgical techniques, such as comparing MIS-TLIF with other minimally invasive procedures or traditional open surgeries in a more systematic way. This limited the ability to clearly discern the relative advantages or disadvantages of each method.

Patient-centered outcomes: The discussion was heavily centered on technical and procedural advancements without sufficient emphasis on patient-centered outcomes such as patient satisfaction, pain management post-surgery and the psychosocial impacts of surgical interventions.

## 5. Conclusions

This comprehensive review underscores the necessity for precise diagnostic and management strategies for LFS, highlighting the role of a multidisciplinary approach and the utility of a unified classification system in enhancing patient outcomes in the face of this condition’s increasing prevalence.

## Figures and Tables

**Table 1:** Studies included in the systematic review.

Study [Ref.]	Study Design	Participant Demographics	Diagnostic Criteria	Interventions	Outcome Measures	Key Findings	Complications
Yamada et al. [13]	Cohort study	A total of 38 patients receiving L5-S1 transforaminal lumbar interbody fusion for L5-S1 foraminal stenosis (FS group) and 60 patients receiving L4-5 decompression and/or fusion for L4-5 intra-spinal canal stenosis (CS group).	MRI and clinical evaluation	MIS-TLIF	-	The prevalence of leg pain was significantly higher in the FS group compared to the CS group (76 vs. 35%). The visual analogue scale for leg pain at rest was also significantly higher in the FS group than in the CS group (6.6 ± 3.1 vs. 1.3 ± 1.9).	None
Kaneko et al. [14]	Case-control study	A total of 77 women of age 50 or more (mean 69.4 years; range 50–83 years) with DLS of 10 degrees or more on a standing posteroanterior radiograph and 19 women aged 50 or more without DLS but with non-specific back pain were included in this study as the control group.	MRI and clinical evaluation	MIS-TLIF, foraminotomy	FH, FW, PDH, P-SAP, cross-sectional FA	In a comparison between the DLS group and the control group, each parameter was smaller in the DLS group, with the greatest difference in FA at L5–S1	None
Liu et al. [15]	Cohort study	A total of 72 patients (17 men and 55 women) with single-level degenerative lumbar spondylolisthesis and unilateral lower extremity symptoms combined with contralateral mild symptoms who underwent MIS-TLIF.	MRI and clinical evaluation	MIS-TLIF	FH, FW, DH, RTP, CSCA measured at surgical and contralateral sites	Unilateral MIS-TLIF can effectively improve contralateral FH, DH, FW, RTP and CSCA. It is not necessary to routinely perform contralateral intervertebral foramen decompression in degenerative lumbar spondylolisthesis with bilateral symptoms when symptoms are mild on one side.	No complications occurred during the follow-up period except for some minor complications.
Lorenc et al. [16]	Cross-sectional study	A total of 99 patients who had a history of back, buttock or leg pain were enrolled in a retrospective study.	MRI and clinical evaluation	None	Cross-sectional FA, DSCSA	Age (*p* < 0.0001), lumbar disc degeneration grade (*p* < 0.016) and DSCSA (*p* < 0.0001) were found to statistically significantly influence the foraminal area (FA).	None
Yan et al. [17]	Cross-sectional study	A total of 25 asymptomatic male volunteers	CT	None	DH, P-SAP, IPV-SAP, SP-IFE	Overall, P-SAP and P-IV decreased and IPV-SAP increased from the entrance to the exit of intervertebral foramen for L3/4-L5S1. DH decreased at entrance slice, middle slice and exit slice for L3/4-L5S1 with age. A significant difference with aging was found only at the middle slice of L3/4 and L4/5 for P-SAP.	None
Chen et al. [18]	Case control study	A total of 190 patients who underwent TLIF	CT	Unilateral TLIF	Radiological parameters including lumbar lordosis, segmental angle, anterior disc height, posterior disc height (PDH), foraminal height (FH), foraminal width and foraminal area (FA) were measured.	The most common cause of contralateral radiculopathy was contralateral foraminal stenosis. Improper unilateral TLIF decreased the PDH, FA and FH, resulting in contralateral radiculopathy.	Two patients underwent revision surgery because of facet subluxation and screw mispositioning. One patient with a hematoma was treated with epidural injection.
Sunday et al. [19]	Cross-sectional study	A total of 250 intervertebral foramina derived from 25 male cadaveric specimens	CT	An osteotomy of the iliac crest was performed to adequately expose the fifth lumbar (L5-S1) foramina.	The foramen height and the foraminal length	The results showed a gradual increase in the foramina height was observed on both the right and left side from L1-L2 to L3-L4 and from then on, decreased progressively towards the L5-S1 level. No statistical difference was noted in the measurements derived (*p* < 0.05).	None
Lee et al. [20]	Cross-sectional study	A total of 96 patients over 60 years old from databases of MR examinations of the lumbar spine performed at the institution in June 2007. There were 35 men (36.5%) and 61 women (63.5%).	MRI	None	Grade 0 indicates normal foramina; grade 1, slight foraminal stenosis and deformity of the epidural fat; grade 2, marked foraminal stenosis; grade 3, advanced stenosis with obliteration of the epidural fat.	The new grading system for foraminal stenosis of the lumbar spine showed nearly perfect interobserver and intraobserver agreement and would be helpful for clinical studies and routine practice.	One limitation was that the grading system was based on sagittal MR morphology without symptomatic correlation.
Lewandrowski et al. [21]	Cohort study	A total of 3560 patients from 168 different MRI imagining center locations around the USA were included. There were 46% male and 51.9% female patients. The remaining 2.1% chose not to identify their gender.	MRI	None	Accuracy, sensitivity, specificity	Deep learning algorithms may be used for routine reporting in spine MRI. There was a minimal disparity among accuracy, sensitivity and specificity, indicating that the data were not overfitted to the training set.	None
Gkasdaris et al. [22]	Cross-sectional study	Patients who underwent CT scanning of their chest and/or abdomen were included. All of them were of Caucasian origin. The chosen individuals underwent CT scanning on their lumbar spine for reasons concerning pathological entities of the gastrointestinal and urinary tract, which did not affect in any morphological way the lumbar vertebrae.	MRI	None	Evaluation of CrFW, CaFW, VH and FH	Age had a negative impact on the height of the elderly due to age-related degenerations and ongoing remodeling.	At the beginning of the study, two individuals were excluded due to gross morphological abnormalities that were visible on their CT images.
Haimoto et al. [23]	Single-center retrospective comparative study	A total of 21 consecutive patients who underwent single-level MFD. Group 1 (7 patients with poor outcomes requiring revision surgery), group 2 (14 patients with good outcomes with no revision surgery required).	MRI and CT	Two surgical procedures for each case: medial foraminotomy or lateral foraminotomy.	Preoperative DW angle, DH, FH	LFS presenting with large DW and lumbar degenerative kyphosis should be excluded from surgical indications for MFD without instrumented fusion, considering the high recurrence rate.	There were no complications associated with the initial surgery with MFD. However, seven patients showed recurrent symptoms with an average recurrence interval of 15.8 months (range, 3–40 months).
Deer et al. [24]	Systematic review	Literature searches yielded nine studies (two RCTs; seven observational studies, four prospective and three retrospective) of minimally invasive spine treatments and one RCT for spacers	MRI and CT	Not specified	Evidence strength, recommendation grade and consensus level using U.S. Preventive Services Task Force criteria.	There was level I evidence for percutaneous image-guided lumbar decompression as superior to lumbar epidural steroid injection and the use of one RCT-supported spacer in a noninferiority study comparing two spacer products currently available.	More commonly, qualitative criteria are used for the diagnosis of LSS, leading to inconsistent inter-reader agreement.
Jeong et al. [25]	Cohort Study	All 99 consecutive patients who underwent unilateral lumbar foraminotomy for LFS were studied between July 2014 and June 2015. There were 45 men (45.5%) and 54 women (54.5%).	MRI and CT	All patients received sufficient conservative treatment and if the pain persisted or it did not subside enough despite conservative treatment, surgical treatment was considered.	A 4-point MRI grading system	An MRI grading system for LFS was thought to be useful as a diagnostic tool for surgery in the lumbar spine. It was less reliable for symptomatic L5–S1 foraminal stenosis than for other levels.	Various clinical factors as well as the MRI grading system are required for surgical decision-making, especially at the L5-S1 region.
Lim et al. [26]	Cross sectional study	Patients who had LFS on conventional MR. The patient group consisted of 33 women and 15 men.	MRI	None	Three morphologic changes (swelling, indentation and tilting angle abnormality)	A 3D MR lumbosacral	None
Uchikado et al. [27]	Retrospective study	Patients with herniated lumbar discs	3D-CT	PELD: transforaminal or interlaminar	Kambin’s triangle	Kambin’s triangle became narrow based on the reduction in the height of the intervertebral disc with the degeneration of intervertebral discs and joints associated with aging.	None
Özer et al. [28]	Cohort study	A total of 115 patients (59 women and 56 men) underwent surgery for LFS.	MRI	Decompression procedure, complete or partial resection of the pedicle, microsurgery or endoscopic techniques.	VAS and ODI scores	This classification helps to determine the optimal treatment. The patients who were operated on according to the classification experienced satisfactory clinical outcomes and low complication rates.	No patients experienced postoperative radiculopathy complications. Only two patients experienced superficial operation site infection and one showed deep wound infection.
Cho et al. [29]	Retrospective review	A total of 33 patients (40 levels) had TLIF and 34 patients (39 levels) had PLIF. The two groups had similar demographic profiles.	CT and MRI	TLIF surgery was performed using either a conventional open technique or a minimally invasive technique. PLIF procedures were performed using a traditional open technique.	FH, SCA. Surgical results were assessed by Odom criteria, VAS and ODI score.	TLIF may induce uneven changes in foraminal morphometry. Cage position may be the major determinant of this result.	One patient complained of contralateral leg pain after open TLIF. The patient was treated medically and fortunately his symptoms disappeared 3 months later.
Zhu et al. [30]	Retrospective cohort study	The patients were divided into two groups according to the cage insertion orientation: the oblique group (o-group, 39 cases) and the transverse group (t-group, 30 cases).	CT and MRI	unilateral transforaminal lumbar interbody fusion (TLIF)	Segmental angle, foraminal height and area	Compared with oblique cage insertion, transverse cage insertion could achieve greater restoration of segmental lumbar lordosis without decreasing contralateral foraminal dimensions	None
Yan et al. [31]	Cross sectional study	There were 25 asymptomatic male volunteers, all of whom underwent lumbar spine CT at the Shanghai East Hospital.	CT	None	Foraminal Height, foraminal width and three different sagittal slices (inside, middle, outside).	Overall, the intervertebral foramen changes occurred in the inner part from middle age to old age. The foraminal height decreased with age in the inside sagittal slice. The foraminal width showed no decrease in each age group or each sagittal plane.	None
Sartoretti et al. [7]	Retrospective cohort study	A total of 101 patients (54 men; 47 women)	MRI	None	Grade A, B, C, D, E, F	The readers found no foramen that could not be described accurately with the updated grading system. Thus, an updated 6-point grading system for LFS was reproducible and comprehensively described LFS as seen on high-resolution MRI.	The grading system was based on static sagittal MR images without symptomatic correlation. Specifically, clinical symptoms may arise only with dynamic changes, such as lumbar extension.

FH, foraminal height; FW, foraminal width; DH, disc height; PDH, posterior disc height; FA, foraminal area; DSCSA, dural sac cross-sectional area; TLIF, transforaminal lumbar interbody fusion; MIS-TLIF, minimally invasive transforaminal lumbar interbody fusion; DLS, degenerative lumbar scoliosis; PLIF, posterior lumbar interbody fusion; PELD, percutaneous endoscopic lumbar discectomy; RCTs, randomized controlled trials; CrFW, cranial foramen width; CaFW, caudal foramen width; VH, vertebral height; SCA, segmental Cobb angle; VAS, visual analog scale; ODI, Oswestry disability index; MFD, microscopic foraminal decompression; P-SAP, pedicle to articular process distance; IPV-SAP, posteroinferior margin of upper vertebrae to articular process distance; SP-IFE, spinous process to intervertebral foramen entrance; LFS, lumbar foraminal stenosis.

**Table 2:** Preoperative-assessment-related studies included in the systematic review.

Study [Ref.]	Lumbar Segment	Preoperative Assessment	Surgical Technique	Anesthesia Type	Hospital Stay (h)
Liu et al. [15]	L3-L4 and L5-S1	The preoperative values were comparable between operative and contralateral sides.	TLIF	General anesthesia	72
Chen et al. [18]	L3-S1	View of the preoperative CT scans and magnetic resonance images is necessary, especially on the contralateral side.	Lumbar fusion	General anesthesia	72
Uchikado et al. [27]	L1–5 levels	The vascular anatomy surrounding the intervertebral foramen is extremely important to prevent complications	Percutaneous endoscopic lumbar discectomy	Local anesthesia	16
Demondion et al. [32]	L5-S1	-	Percutaneous endoscopic lumbar discectomy	General	12

TLIF, transforaminal lumbar interbody fusion; h, hours.

**Table 3:** Classification-system-related studies included in the systematic review.

Study [Ref.]	Type of Study	Measurement Technique	Classification System	Sample Size	Key Diagnostic Criteria	Clinical Relevance
Kaneko et al. [14]	Retrospective	Computed tomography	Pedicle-to-Pedicle Method	77	Foraminal height, FW	Clinical symptoms
Liu et al. [15]	Retrospectively analyzed	C-arm fluoroscopy.	Pedicle-to-Pedicle Method	72	Foraminal height, FW, disc height	Clinical symptoms and imaging finding
Lorenc et al. [16]	Observational study	A 1.5 T MRI	Spine-related pain	90	Intervertebral foramen	Symptoms and the value of MRI
Chen et al. [18]	Retrospectively reviewed	CT scans and MRI	Nerve block	190	Foraminal height, FW and foraminal area	Correlation to clinical symptoms
Sunday et al. [19]	Retrospective	Post hoc test	Not specified	250	Foraminal heights	Adequate clinical evaluation and measurements using imaging
Haimoto et al. [23]	Retrospective comparative study	MRI and CT	Pathogenesis of LFS	21	Foraminal heigh	Clinical symptoms of lumbar radiculopathy
Deer et al. [24]	Systematic review	MRI, CT or fluoroscopy.	Not specified	11	Transforaminal	Clinical features that are commonly attributed to this include lower back pain, radicular leg pain and neurogenic Claudication.
Jeong et al. [25]	Retrospective	A 4-point MRI grading system	Epidural fat obliteration	99	MRI grading system for LFS	Pain intensity and radiologic findings
Lim et al. [26]	Retrospective	MR imaging was performed with a 1.5-T scanner		48	The signal intensity of fat was completely suppressed.	The swelling, indentation and tilting angle abnormality of nerve roots in the foraminal
Özer et al. [28]	retrospective studies	None	stable and unstable stenosis	115	Foraminal height	Root irritation becomes more prominent both clinically and radiologically
Cho et al. [29]	Retrospective review	CT	Pedicle-to-Pedicle Method	67	Disc height, foraminal height and segmental Cobb angle	Directly correlated with symptom severity
Zhu et al. [30]	Retrospective cohort study	Impax PACS	Not described	69	Segmental lumbar lordosis without decreasing contralateral foraminal dimensions	Asymptomatic
Comer et al. [33]	Retrospective analysis	Computed tomographic	Pedicle-to-Pedicle Method	67	Foraminal height and segmental Cobb angle	Directly correlated with symptom severity
Hutchins et al. [34]	Systematic search	MRI	No described	23 papers	-	correlation to clinical symptoms

MRI, magnetic resonance images; FW, foraminal width.
